# Perfusion Computed Tomography for Assessing Pancreas Graft Volumetric Perfusion After Simultaneous Pancreas and Kidney Transplantation

**DOI:** 10.3390/diagnostics14212361

**Published:** 2024-10-23

**Authors:** Ilya V. Dmitriev, Rustam Sh. Muslimov, Yuriy A. Anisimov, Svetlana P. Shchelykalina, Elena V. Grigorieva, Igor O. Shchekoturov, Natalya S. Serova, Sergey K. Ternovoy

**Affiliations:** 1 Sklifosovsky Research Institute for Emergency Medicine, 129090 Moscow, Russia; abaevr@mail.ru (R.S.M.); ya@anisimov86.ru (Y.A.A.); 2 Department of Medical Cybernetics and Computer Science MBF, Pirogov Russian National Research Medical University, 117997 Moscow, Russia; svetlanath@inbox.ru; 3 Radiological Department of Clinical Medical Center, FSBEI HE «ROSUNIMED» of MOH of Russia, 127473 Moscow, Russia; iara333@yandex.ru; 4 Department of Radiology and Radiotherapy, I.M. Sechenov First Moscow State Medical University, 119991 Moscow, Russia; samaramail@bk.ru (I.O.S.); dr.serova@yandex.ru (N.S.S.); prof_ternovoy@list.ru (S.K.T.)

**Keywords:** pancreas transplantation, pancreas graft revascularization, perfusion computed tomography

## Abstract

**Background:** There is paucity of data in the available medical literature regarding the parameters of the volumetric perfusion of pancreas grafts. **Methods:** From 5 February 2016 to 23 December 2021, we performed perfusion computed tomography in 41 patients at different times after simultaneous pancreas and kidney transplantation. The study group consisted of 18 men (44%) and 23 women (56%) with a long history of type 1 diabetes mellitus complicated by terminal chronic renal failure. The results of the perfusion computed tomography of the pancreas graft were studied, and the effects of post-transplantation timing and graft revascularization peculiarities on volumetric perfusion parameters were evaluated. **Results:** The median arterial blood flow, arterial blood volume, and permeability of the pancreas graft were 115.1 [99.7;130.3] mL/100 mL/min, 46.7 [37.4;56.9] mL/min, and 8.6 [4.1;11.4] mL/100 mL/min, respectively. No statistically significant differences in the averaged perfusion values were found in the head, body, and tail of the pancreas graft. The post-transplantation timing and the number of arteries involved in graft revascularization did not have a significant effect on the volumetric perfusion of the graft. **Conclusion:** The volumetric perfusion results of the pancreas graft correspond to those obtained in the study of pancreatic perfusion in healthy participants.

## 1. Introduction

At the current stage of clinical transplantation, the average half-life of pancreas grafts (PGs) is 16.7 years, which corresponds to the half-life of kidney grafts from deceased donors and is the longest among extrarenal organs [1]. PG dysfunction in the long-term post-transplantation period is often due to graft sclerosing processes and microcirculation pathology, both immunologic and non-immunologic [2,3]. Hence, the assessment of the PG volumetric intra-organ perfusion is of fundamental importance in pancreas transplantation. The arterial anatomy of the pancreas is usually assessed using various X-ray diagnostics: ultrasonic color Doppler imaging, computed tomography (CT), magnetic resonance imaging (MRI), and selective angiography. The resolution of the images obtained using these methods allows the visualization of vessels with a diameter of 1 mm and larger; therefore, they are not useful for the assessment of the capillary bed. The problem of a pancreatic capillary perfusion assessment can be solved through perfusion computed tomography (PCT): a method of the bolus contrast enhancement of the organ enabling the subsequent assessment of contrasted blood flow through all of the branches of its arteries and veins and its capillary beds. The principle of obtaining perfusion maps from different manufacturers is similar, but the existing differences in the software used to generate perfusion maps, the width of the CT scanner detector, the voltage and current on the X-ray tube, and the differences in the parameters used to estimate blood flow impose limitations on the comparison of values obtained on different CT scanners. Therefore, CT perfusion standards will be different on different devices. Additionally, various methods of primary data collection and post-processing are used. There are several options for data deconvolution. For example, with delay-sensitive deconvolution, the perfusion curve is constructed from the moment the contrast enters the reference artery, without taking into account the degree of density increase in the vessel lumen. With delay-intensive deconvolution, the perfusion curve is constructed from the moment the threshold density values are reached in the lumen of the reference artery. This means that a number of deconvolution options have an error in the case of when the blood flow in the feeding artery is impaired, for example, against the background of atherosclerosis, tumor invasion, and thrombosis. The choice of deconvolution options depends on the manufacturer and the model of the device. So, it is recommended that dynamic studies be performed on the same CT scanner to reliably assess changes in blood flow values.

In 1995, Miles et al. described the methodology and evaluated pancreas PCT parameters in patients with different physiological and pathological conditions [4]. In addition, the article presented the parameters of native pancreas perfusion and post-transplantation PG perfusion in one patient with diabetes mellitus. In the following years, reviews [5,6] and original articles were published on native pancreatic perfusion in healthy participants [7,8,9,10,11,12], in participants with inflammatory [8,9,12,13,14] and oncologic lesions of the pancreas [14,15,16,17,18], and in participants with several other diseases [19,20]. Despite the high potential of PCT for an objective assessment of PG volumetric perfusion with high spatial and temporal resolution, there is limited application of this method, owing to differences in the technical parameters of data acquisition and post-processing, including the use of multivendor devices, as well as the lack of unified reference values for the volumetric perfusion of pancreas grafts.

Little attention has been paid to the study of PG perfusion. Only one study, dedicated to a pancreas transplantation method with isolated perfusion via the splenic artery system, reported on PG PCT [21,22]. Hence, this study was conducted to fill the gap in PG perfusion data.

## 2. Materials and Methods

During the period from 5 February 2016 to 23 December 2021, we performed PCT in 41 patients with functioning kidney and pancreas grafts at different times after simultaneous pancreas and kidney transplantation (SPKT). SPKTs were performed during the period from 11 January 2008 to 23 November 2021. There was a retrospective–prospective study design.

### 2.1. Recipients

The pool of recipients consisted of 18 men (44%) and 23 women (56%) with a median age of 34 [31;39] years and a median body mass index of 20.7 [19.4;23.4] kg/m^2^. The patients had an early onset and prolonged course of diabetes mellitus (DM); the median age of DM manifestation was 11 [7;14] years, and the duration of DM at the time of transplantation was 24 [20;29] years. Thirty-eight recipients had received renal replacement therapy: twenty-nine (71%) through hemodialysis and nine (22%) through peritoneal dialysis. The median duration of renal replacement therapy was 2 [1;3] years. Only three patients underwent pre-dialysis transplantation.

### 2.2. Donors

Organ explantation, prior to grafting, was performed as part of a multiorgan harvesting procedure in patients with confirmed brain death. In most cases, this was due to craniocerebral trauma (*n* = 25, 61%). In a smaller number of cases, this was due to acute cerebrovascular accidents (*n* = 16, 39%). Most donors were men (*n* = 34, 83%), and the median age of the donors was 28 [25;32] years.

### 2.3. Pancreas Transplantation Technique

The majority of patients underwent SPKT with retroperitoneal localization of the PG (*n* = 37, 90%). Only four patients (10%) had intraperitoneal localization of the PG. Thirty-four patients (83%) underwent PG transplantation after preliminary arterial reconstruction using Y-grafts, and seven patients (17%) had isolated perfusion via the splenic artery system only. In most cases (*n* = 39, 95%), venous drainage was directed into the inferior vena cava system (systemic venous drainage), while in two cases (5%), it was directed into the portal vein system. Exocrine drainage of the PG was ensured by duodeno-duodenal (30 recipients/73%) or duodeno-jejunal anastomosis (11 recipients/27%). The median durations of kidney graft and PG preservation were 8 [6.5–9] and 9 [8–10.5] hours, respectively.

### 2.4. Immunosuppression

Patients received triple immunosuppressive therapy (IST) including calcineurin inhibitors (tacrolimus and cyclosporine), antimetabolites (mycophenolate mofetil and mycophenolic acid), and glucocorticoids (prednisolone). Tacrolimus was the most commonly used calcineurin inhibitor in basic IST (*n* = 39, 95.1%). As an induction IST, monoclonal antibodies (basiliximab) were used in 28 patients (68.3%), and polyclonal antibodies (rabbit antithymocyte globulin or equine antithymocyte globulin) were applied in 13 recipients (31.7%).

### 2.5. PCT Methodology

**Figure 1:**
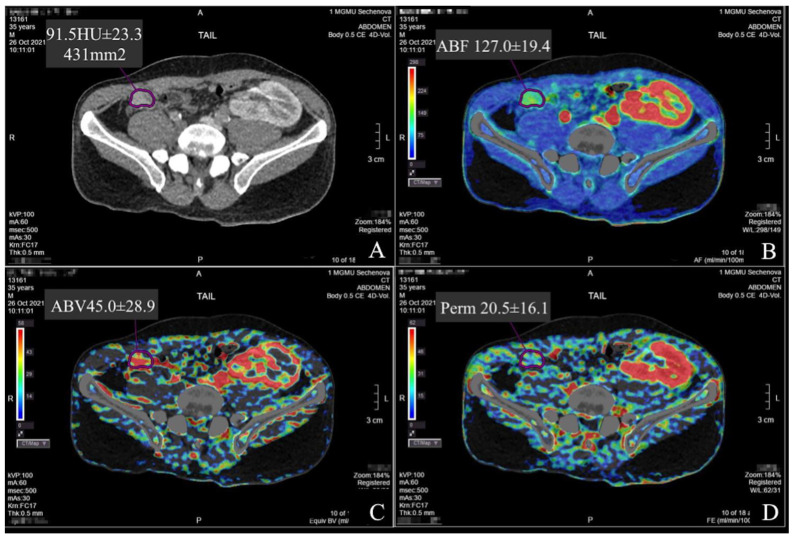
Volumetric blood flow indicators in the tail of the pancreas graft. (**A**) Conventional CT. The ROI is highlighted with a contour—pancreas graft; (**B**) arterial blood flow (ABF); (**C**) arterial blood volume (ABV); (**D**) permeability (Perm).

Selected parameters of the PG intra-organ hemodynamics were evaluated by PCT on a 640-slice Aquilion One CT scanner (Toshiba, Japan). A low-dose protocol with intermittent abdominal tumor perfusion scanning was used. The data acquisition period was 100 s. The data acquisition specifications were as follows: tube voltage—100 kV, tube current—60 mA, slice thickness—0.5 mm, rotation time—0.5 s, scan area width—160 mm, and matrix—512 × 512 pixels. Dynamic studies were performed without breath-holding, after a short pre-briefing of patients to prevent forced inhalation. Yopromide (Ultravist, Bayer Pharma AG, D-13342, Berlin, Germany), with an iodine concentration of 370 mg/mL, was used as a contrast medium. Contrast medium was injected in an amount of 0.5 mL per kilogram of the patient’s body weight at a rate of 6–7 mL/s. The median volume of the injected contrast medium was 30 [26.5;33.5] mL. The post-processing and analysis of the data array obtained were performed by the maximum slope method on the Vitrea workstation (Vital Inc., Minnetonka, MN, USA). The PCT data of all the patients were analyzed by two radiologists independently of each other. Radiologist 1: the leading researcher of the Department of Radiological Diagnostics, PhD, professional experience—15 years; they are an expert in the field of cardiovascular imaging, imaging in transplantation, and emergency conditions. Radiologist 2: a member of the European Society of Radiologists, PhD, professional experience—8 years; they are an expert in the field of perfusion studies of the liver, kidneys, skin, and muscle autografts. The study was observer-blinded: the radiologists did not have information on the postoperative period, results of other clinical investigations, and treatment outcomes. A reference arterial and parenchymal input curve was obtained by placing regions of interest (ROIs) in the aorta and pancreatic tissue, followed by blood vessel segmentation and the calculation of perfusion maps. ROIs were placed in the normal parenchyma of the head, body, and tail of the pancreas. Vascular structures were avoided when placing ROIs. The area of the ROIs was standardized for both radiologists at 15 mm^2^. Perfusion parameters, such as arterial blood flow (ABF), arterial blood volume (ABV), and permeability, were analyzed on these maps (Figure 1).

First, the volumetric perfusion parameters in the head, body, and tail of the PG were compared. Thereafter, the patients were divided into three groups to ensure the reliable assessment of the effects of post-transplantation timing on PG volumetric perfusion parameters. Group I included patients with the study timing up to 1 year post transplantation (*n* = 15, 37%), Group II was 1 to 3 years (*n* = 14, 34%), and Group III was more than 3 years (*n* = 12, 29%). These groups showed no statistically significant differences (*p* < 0.05) in parameters related to the recipients, donors, and surgical techniques used.

To reliably assess the possible effect of the number of PG perfusion-critical arteries (isolated blood flow via the splenic artery system or arterial reconstruction using a Y-graft) on the volumetric perfusion parameters, the patients were divided into two groups: I_ISABS_ and II_Y-graft_. The I_ISABS_ group comprised seven patients, and the II_Y-graft_ group consisted of thirty-four patients. These groups also showed no statistically significant differences (*p* < 0.05) in recipient-related and donor-related factors and in surgical technique parameters.

### 2.6. Estimation of Effective Radiation Dose (Radiation Exposure)

The effective radiation dose E was calculated by the formula E = DLP *Edlp, where DLP (dose length product) was the dose absorbed during the whole CT study with the scan length considered. Edlp was the normalized effective dose for a specific study area. According to the “European Guidelines on Quality Criteria for Computed Tomography”, Edlp for the abdominal cavity is 0.015.

### 2.7. Statistical Analysis

The statistical analysis of the data was performed using Statistica for Windows v. 10.0, (StatSoft Inc., Tulsa, OK, USA) software package. The normality of distribution was checked with the Shapiro–Wilk test. The following tests were used to compare the quantitative characteristics of different groups: the Mann–Whitney test for two independent groups, the Wilcoxon test for two related groups, the Kruskal–Wallis test for three independent groups, and the Friedman test for three related groups. Differences were considered statistically significant at *p* < 0.05 for single comparisons and *p* < 0.017 for pairwise comparisons, with Bonferroni’s adjustment considered.

## 3. Results

### 3.1. The Volumetric Perfusion Results of the Pancreas Graft

The medians of the patients’ PCT results, obtained by both radiologists, are presented in Table 1.

No statistically significant differences were found between the PCT results obtained by both radiologists: ABF 114 [98.8;130.3] vs. 116.3 [103.8;128.1], *p* = 0.18, ABV 47 [37.2;56.9] vs. 46.1 [38.2;56.9], *p* = 0.24 and Perm 8.5 [4.1;11.4] vs. 8.6 [4.2;11.4], *p* = 0.12.

The medians of the PCT results in different parts of the PG are presented in Table 2.

Statistically significant differences were noted in the ABF values obtained by Radiologist 1: when comparing the ABF values in the body and tail (118.2 [101.9;134.3] mL/100 mL/min vs. 110.7 [96.5;129.8] mL/100 mL/min, *p* = 0.016) with those in the head and tail (116.9 [97.9;127.8] mL/100 mL/min vs. 110.7 [96.5;129.8] mL/100 mL/min, *p* = 0.01), the tail value was smaller. No statistically significant differences were noted in the volumetric perfusion values obtained by Radiologist 2 (*P_ABF_*_(h-b-t)_ = 0.84, *P_ABV_*_(h-b-t)_ = 0.39, *P_Perm_*_(h-b-t)_ = 0.67). Comparison of the values obtained by the two radiologists showed that the ABF and ABV tail measurements were poorly reproduced: the values obtained by Radiologist 2 were significantly larger than those obtained by Radiologist 1 (ABF(t)_R1_ 110.7 [96.5;129.8] mL/100 mL/min vs. ABF(t)_R2_ 118.4 [101.2;131.2] mL/100 mL/min, *p* = 0.003 and ABV(t)_R1_ 42.4 [35.5;54] mL/min vs. ABV(t)_R2_ 44.7 [38.9;59.3] mL/min, *p* = 0.036). However, no other statistically significant differences were noted, including no differences in the averaged values in the parts of the PG.

### 3.2. The Impact of the Timing of Pancreas Transplantation on the Volumetric Perfusion of Pancreas Graft

The comparative analysis of the PCT data obtained by each radiologist for the three groups of patients, based on post-SPKT timing, is presented in Table 3 and Table 4 and Figure S1 (Supplemental Materials).

No statistically significant differences were found when comparing the values obtained by both radiologists for each group (Group 1: P_ABF R1-ABF R2_ = 0.21, P_ABV R1-ABV R2_ = 0.23, P_Perm R1-Perm R2_ = 0.29; Group 2: P_ABF R1-ABF R2_ = 0.38, P_ABV R1-ABV R2_ = 0.67, P_Perm R1-Perm R2_ = 0.81; and Group 3: P_ABF R1-ABF R2_ = 0.67, P_ABV R1-ABV R2_ = 0.32, P_Perm R1-Perm R2_ = 0.12) (Table 4).

### 3.3. The Impact of the Revascularization Peculiarities on the Volumetric Perfusion of Pancreas Graft

The data presented in Table 5 and Table 6 and Figure S2 (Supplemental Materials) were obtained during the assessment of the possible effect of revascularization peculiarities.

Surprisingly, the ABF values for the I_ISABS_ group were higher than those of the II_Y-graft_ group (Radiologist 1: ABF I_ISABS_ 128.1 [110.7;154] mL/100 mL/min vs. ABF II_Y-graft_ 113 [97.8;127.6] mL/100 mL/min; Radiologist 2: ABF I_ISABS_ 126.7 [112.3;146.4] mL/100 mL/min vs. ABF II_Y-graft_ 115 [101.8;127.8] mL/100 mL/min). However, no statistically significant differences were noted between the PG volumetric perfusion parameter results in cases with isolated revascularization through the splenic artery compared to those with revascularization through the superior mesenteric and splenic arteries using a Y-graft ((Radiologist 1: P_ABF I ISABS-II Y-graft_ = 0.15, P_ABV I ISABS-II Y-graft_ = 0.82, and P_Perm I ISABS-II Y-graft_ = 0.89; Radiologist 2: P_ABF I ISABS-II Y-graft_ = 0.28, P_ABV I ISABS-II Y-graft_ = 0.59, and P_Perm I ISABS-II Y-graft_ = 0.94) (Table 5) and (Group I_ISABS_: P_ABF R1-ABF R2_ = 0.6, P_ABV R1-ABV R2_ = 0.46, and P_Perm R1-Perm R2_ = 0.69; Group II_Y-graft_: P_ABF R1-ABF R2_ = 0.06, P_ABV R1-ABV R2_ = 0.31, and P_Perm R1-Perm R2_ = 0.07) (Table 6)).

### 3.4. The Impact of Donor-Related, Recipient-Related and Surgical Factors on the ABF Values of the Pancreas Graft

The analysis showed significant differences in the PG ABF values of the studied recipients. These values were used as a basis to divide the patients into three groups: Group I_perf_ consisted of patients with ABF values above 120 mL/100 mL/min, Group II_perf_ from 100 to 120 mL/100 mL/min, and Group III_perf_ below 100 mL/100 mL/min. The analysis and comparison of recipient-related, donor-related, and surgical factors in Groups I_perf_, II_perf_, and III_perf_ showed that none of them had a statistically significant effect on the degree of pancreas graft perfusion.

### 3.5. Effective Radiation Dose (Radiation Exposure)

Radiation doses during the perfusion study ranged between 7.2 and 24.3 mSv; the mean effective dose was 13.7 [12.2–16.1] mSv.

## 4. Conclusions

The single-stage assessment of the entire pancreas graft has become possible with the advent of advanced equipment with a wide detector, which allows the performance of volumetric studies up to 16 cm in length. Despite the potential of PCT for the objective assessment of volumetric perfusion of PGs with high spatial and temporal resolution, there is limited application of this method because of the differences in the technical parameters of data acquisition and post-processing, including the use of multivendor devices. In addition, the lack of unified reference values of pancreatic volumetric perfusion for various pancreas-related diseases, and for healthy participants, hinders the wide and routine use of the method.

We conducted a pilot study of PG perfusion using the PCT method in 41 patients after SPKT as there is still a dearth of data on this problem in the publicly available medical literature. According to D. T. Doherty et al., PCT is a promising method of PG imaging, which may improve the quality of PG volumetric perfusion assessment and assist in the diagnosis of early vascular and later immunologic complications, as well as the degree of total fibrosis in the PG [23].

This study of the volumetric perfusion of the PG parenchyma using PCT in 41 patients with functioning kidney and pancreas grafts at different times after SPKT showed the following averaged results: ABF 115.1 [99.7;130.3] mL/100 mL/min, ABV 46.7 [37.4;56.9] mL/min, and permeability 8.6 [4.1;11.4] mL/100 mL/min.

**Figure 2:**
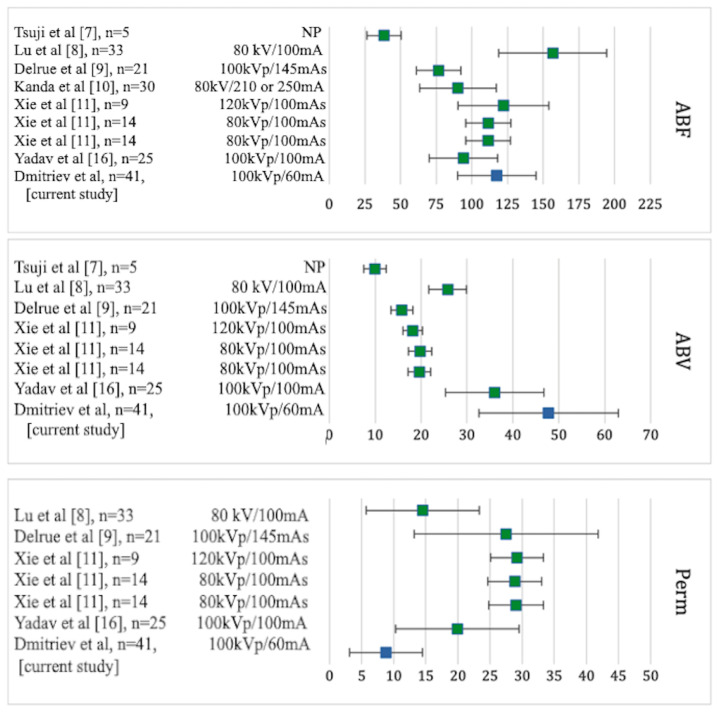
Pancreatic volumetric perfusion parameters (ABF, ABV, and permeability) in healthy participants who formed the control groups in the respective studies (highlighted in green) and the PG results obtained in the present study (highlighted in blue) (M and SD) [7,8,9,10,11,16].

A comparison of the results of our perfusion study with the data obtained in the study of perfusion in healthy participants who formed the control group in other studies [7,8,9,10,11,16] is presented in Figure 2.

ABF values detected in the study were comparable to ABF values in healthy subjects; however, the ABV and Perm values differed significantly, which can be due to intraoperative ischemia–reperfusion injury as well as chronic immunosuppression.

No statistically significant differences were noted when comparing the average values of the volumetric perfusion parameters for the head, body, and tail of the PG, as obtained by the two radiologists. Occasional statistically significant differences between the intra-organ volumetric perfusion parameter values obtained by these radiologists were probably caused by the different levels of value determination, which confirms the necessity of analyzing the values averaged from 3 to 5 sites.

No statistically significant differences between PCT results were noted in patients divided into groups based on post-SPKT timing. This may indicate that the perfusion of the organ does not change significantly with time but depends on the initial parameters; however, this warrants further study.

Patients with isolated PG perfusion via the splenic artery system, who underwent technically successful SPKT, showed a richer graft perfusion; however, statistically significant differences were not noted when compared with the results of the patients with PG perfusion via the superior mesenteric and splenic arteries.

Because of the radiation exposure the common use of perfusion CT may have a limitation. The extra radiation dose is one of the major problems with the CT perfusion method. The radiation dose during the perfusion study was comparable to the mean standard radiation exposure for a three-phase CT examination of the abdominal cavity, which was 18.3 mSv [24].

### Limitations of the Study

The retrospective nature of the study inherently limits the ability to establish causality. Such designs are more prone to biases and confounding factors that cannot be controlled as effectively as in prospective studies. Given the study’s retrospective design and the specific patient population from a single geographic location, the results may not be generalizable to all simultaneous pancreas–kidney recipients. The study may not have controlled for all potential confounding factors, such as variations in immunosuppressive therapy regimens, patient comorbidities, and lifestyle factors that could influence the pancreas graft volume blood supply. Pancreas graft volumetric perfusion parameters in recipients with isolated revascularization through the splenic artery only were conducted on a limited subset of patients (7 out of 41), which may not provide a comprehensive view of the microcirculatory bed of the pancreas graft with isolated splenic artery blood supply. This small sample size limits the generalizability of the findings regarding the volume blood supply in these recipients. Volumetric perfusion parameters of the pancreas graft were evaluated exclusively in patients with functioning grafts and were not evaluated in patients with lost pancreas graft function, in patients with active pancreas graft rejection, and in patients with a histologically verified toxicity of calcineurin inhibitors. These limitations restrict the ability to generalize these results to all pancreas grafts. Among other things, the authors did not assess the correlation between pancreas graft volume blood supply parameters and markers of pancreatic endocrine function.

## Figures and Tables

**Table 1:** Pancreas graft perfusion computed tomography data.

	Radiologist 1	Radiologist 2	*p* *
ABF, mL/100 mL/min	114 [98.8;130.3]	116.3 [103.8;128.1]	0.18
ABV, mL/min	47 [37.2;56.9]	46.1 [38.2;56.9]	0.24
Perm, mL/100 mL/min	8.5 [4.1;11.4]	8.6 [4.2;11.4]	0.12

ABF—arterial blood flow, ABV—arterial blood volume, Perm—permeability, *—Wilcoxon test.

**Table 2:** PCT data in parts of the PG.

Parameter	Value	*p* * (h-b-t)	*p* ** (h-b)	*p* ** (b-t)	*p* ** (h-t)	*p* ** (r1–r2)
Radiologist 1						
ABF (h), mL/100 mL/min	116.9 [97.9;127.8]	0.026	0.4	0.016 ^+^	0.01 ^+^	0.96
ABF (b), mL/100 mL/min	118.2 [101.9;134.3]					0.81
ABF (t), mL/100 mL/min	110.7 [96.5;129.8]					0.003 ^+^
ABV (h), mL/min	49.6 [36.7;57]	0.16	0.064	0.38	0.046	0.64
ABV (b), mL/min	47.2 [33.9;55.2]					0.52
ABV (t), mL/min	42.4 [35.5;54]					0.036 ^+^
Perm (h), mL/100 mL/min	8.4 [4.1;11.3]	0.8	0.92	0.12	0.37	0.55
Perm (b), mL/100 mL/min	8.3 [4.1;12]					0.99
Perm (t), mL/100 mL/min	8.5 [3.6;11.4]					0.11
Radiologist 2						
ABF (h), mL/100 mL/min	116.5 [101.1;131.2]	0.84	0.25	0.61	0.24	0.96
ABF (b), mL/100 mL/min	117 [99.9;139]					0.81
ABF (t), mL/100 mL/min	118.4 [101.2;131.2]					0.003 ^+^
ABV (h), mL/min	47.2 [39.3;55]	0.39	0.18	0.94	0.35	0.64
ABV (b), mL/min	45.1 [37.7;57.8]					0.52
ABV (t), mL/min	44.7 [38.9;59.3]					0.036 ^+^
Perm (h), mL/100 mL/min	9 [4.1;11.2]	0.67	0.77	0.21	0.32	0.55
Perm (b), mL/100 mL/min	8.6 [4.4;12.1]					0.99
Perm (t), mL/100 mL/min	8.1 [3.8;11.4]					0.11

ABF—arterial blood flow, ABV—arterial blood volume, Perm—permeability, h—head, b—body, t—tail; r1—radiologist 1, r2—radiologist 2; * Friedman test, ** Wilcoxon test, ^+^—statistically significant differences.

**Table 3:** PCT data for the three groups of patients based on post-SPKT timing.

	Group I (*n* = 15)	Group II (*n* = 14)	Group III (*n* = 12)	*p* *
Radiologist 1				
ABF, mL/100 mL/min	114.9 [98.8;130.3]	123.1 [105.9;153.7]	106.3 [95.6;128.5]	0.37
ABV, mL/min	47 [33.6;56.9]	45.3 [37.4;52.1]	48.4 [33.7;61.9]	0.92
Perm, mL/100 mL/min	5.1 [3.7;10.6]	10.4 [8.4;11.3]	9 [2.7;11.9]	0.3
Radiologist 2				
ABF, mL/100 mL/min	116.3 [108.9;127.8]	123.6 [104.2;139.2]	107.9 [95.6;129.9]	0.32
ABV, mL/min	46.1 [38;61]	44.7 [38.2;52.1]	48.1 [36.3;60.2]	0.85
Perm, mL/100 mL/min	7.4 [3.9;11.2]	10.1 [8.4;11.4]	9.3 [3.2;12.9]	0.48

ABF—arterial blood flow, ABV—arterial blood volume, Perm—permeability, *—Kruskal–Wallis test.

**Table 4:** PCT data for the groups based on post-SPKT timing.

Group	Radiologist	ABF, mL/100 mL/min	*p* *	ABV, mL/min	*p* *	Perm, mL/100 mL/min	*p* *
I (*n* = 15)	1	114.9 [98.8;130.3]	0.21	47 [33.6;56.9]	0.23	5.1 [3.7;10.6]	0.29
	2	116.3 [108.9;127.8]		46.1 [38;61]		7.4 [3.9;11.2]	
II (*n* = 14)	1	123.1 [105.9;153.7]	0.38	45.3 [37.4;52.1]	0.67	10.4 [8.4;11.3]	0.81
	2	123.6 [104.2;139.2]		44.7 [38.2;52.1]		10.1 [8.4;11.4]	
III (*n* = 12)	1	106.3 [95.6;128.5]	0.67	48.4 [33.7;61.9]	0.32	9 [2.7;11.9]	0.12
	2	107.9 [95.6;129.9]		48.1 [36.3;60.2]		9.3 [3.2;12.9]	

*—Wilcoxon test.

**Table 5:** Comparative analysis of PG volumetric perfusion parameter values in the groups based on the number of revascularization-critical arteries.

	Group I_ISABS_, *n* = 7	Group II_Y-graft_, *n* = 34	*p* *
Radiologist 1			
ABF, mL/100 mL/min	128.1 [110.7;154]	113 [97.8;127.6]	0.15
ABV, mL/min	40.6 [33.6;65.6]	47.1 [37.2;56.9]	0.82
Perm, mL/100 mL/min	10.6 [3.7;11.8]	8.5 [4.1;11.4]	0.89
Radiologist 2			
ABF, mL/100 mL/min	126.7 [112.3;146.4]	115 [101.8;127.8]	0.28
ABV, mL/min	40.6 [32.8;67.1]	46.1 [40.9;56.9]	0.59
Perm, mL/100 mL/min	10.8 [3.7;11.7]	8.6 [4.2;11.4]	0.94

*—Mann–Whitney test.

**Table 6:** Comparative analysis of pancreas graft volumetric perfusion parameter values obtained by both radiologists for the I_ISABS_ and II_Y-graft_ groups.

Group	Radiologist	ABF, mL/100, mL/min	*p* *	ABV, mL/min	*p* *	Perm, mL/100 mL/min	*p* *
I_ISABS_ (*n* = 7)	1	128.1 [110.7;154]	0.60	40.6 [33.6;65.6]	0.46	10.6 [3.7;11.8]	0.69
	2	126.7 [112.3;146.4]		40.6 [32.8;67.1]		10.8 [3.7;11.7]	
II_Y-graft_ (*n* = 34)	1	113 [97.8;127.6]	0.06	47.1 [37.2;56.9]	0.31	8.5 [4.1;11.4]	0.07
	2	115 [101.8;127.8]		46.1 [40.9;56.9]		8.6 [4.2;11.4]	

*—Wilcoxon test.

## Data Availability

The raw data supporting the conclusions of this article will be made available by the authors on request.

## Supplementary Materials

The following supporting information can be downloaded at: https://www.mdpi.com/article/10.3390/diagnostics14212361/s1, Figure S1: ABF values obtained by both radiologists for the groups based on post-SPKT timing. Figure S2: ABF values obtained by both radiologists for the I_ISABS_ and II_Y-graft_ groups.

## Abbreviations

ABF	arterial blood flow.
ABV	arterial blood volume.
CT	computed tomography.
DLP	dose length product.
DM	diabetes mellitus.
ISABS	isolated splenic artery blood supply.
MRI	magnetic resonance imaging.
PCT	perfusion computed tomography.
PERM	permeability.
PG	pancreas graft.
ROI	region of interest.
SPKT	simultaneous pancreas and kidney transplantation.
