# Supplementary material for: Perfusion Computed Tomography for Assessing Pancreas Graft Volumetric Perfusion After Simultaneous Pancreas and Kidney Transplantation
Source: Diagnostics (Basel). 2024 Oct 23;14(21):2361. doi: 10.3390/diagnostics14212361 (PMC11545586; doi:10.3390/diagnostics14212361)
Supplement: Supplementary file 1 [file diagnostics-14-02361-s001.zip › diagnostics-3139382-supplementary.pdf]

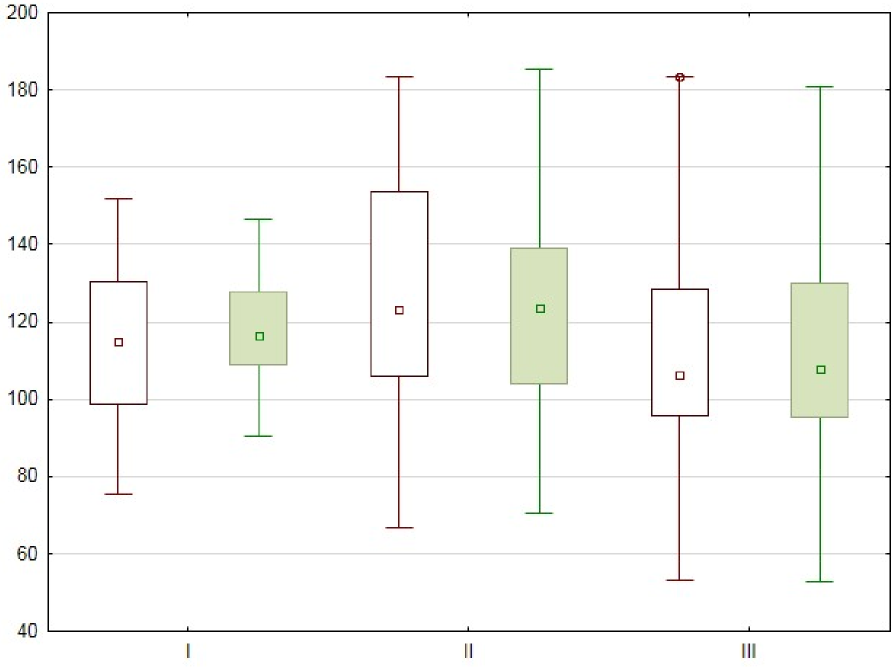

S1. ABF values obtained by both radiologists for the groups based on post-SPKT timing. ◻ Radiologist 1, ◻ Radiologist 2, ☐ median, rectangle – 25–75 %, segment – min–max, ○ outliers

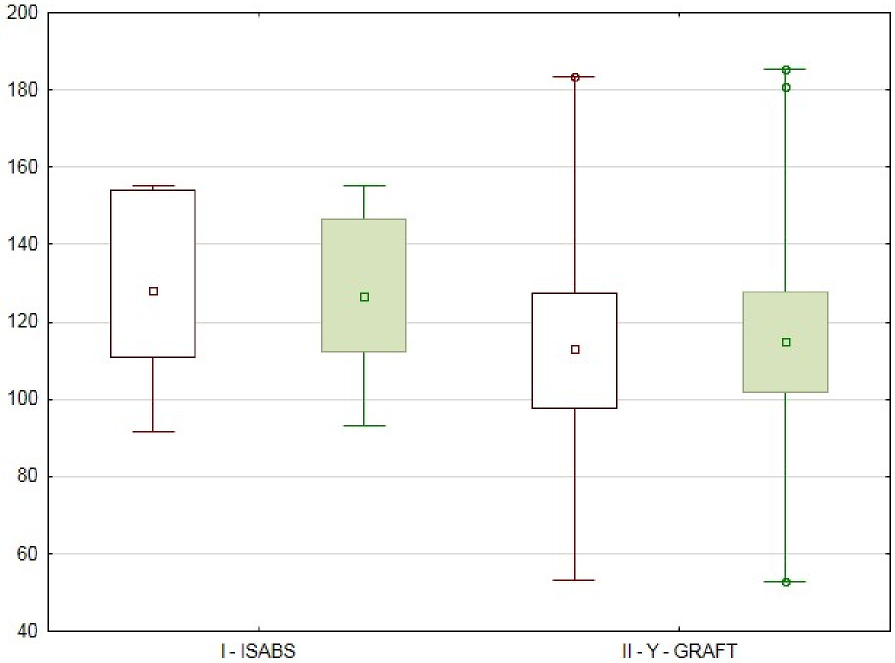

S2. ABF values obtained by both radiologists for the I_{ISABS} and $II_{Y\text{-}graft}$ groups. ◻ Radiologist 1, ◻ Radiologist 2, ☐ median, rectangle – 25–75 %, segment – min–max, ○ outliers
